# Silver: Forging almost Gold Standard Datasets

**DOI:** 10.3390/genes12101523

**Published:** 2021-09-28

**Authors:** Farhad Maleki, Katie Ovens, Ian McQuillan, Anthony J. Kusalik

**Affiliations:** 1 Augmented Intelligence & Precision Health Laboratory, Institute of the McGill University Health Centre, McGill University, Montreal, QC H4A 3S5, Canada; katie.ovens@mail.mcgill.ca; 2 Department of Computer Science, University of Saskatchewan, Saskatoon, SK S7N 5C9, Canada; mcquillan@cs.usask.ca (I.M.); kusalik@cs.usask.ca (A.J.K.)

**Keywords:** gene set analysis, synthetic data, sensitivity, specificity

## Abstract

Gene set analysis has been widely used to gain insight from high-throughput expression studies. Although various tools and methods have been developed for gene set analysis, there is no consensus among researchers regarding best practice(s). Most often, evaluation studies have reported contradictory recommendations of which methods are superior. Therefore, an unbiased quantitative framework for evaluations of gene set analysis methods will be valuable. Such a framework requires gene expression datasets where enrichment status of gene sets is known *a priori*. In the absence of such gold standard datasets, artificial datasets are commonly used for evaluations of gene set analysis methods; however, they often rely on oversimplifying assumptions that make them biased in favor of or against a given method. In this paper, we propose a quantitative framework for evaluation of gene set analysis methods by synthesizing expression datasets using real data, without relying on oversimplifying or unrealistic assumptions, while preserving complex gene–gene correlations and retaining the distribution of expression values. The utility of the quantitative approach is shown by evaluating ten widely used gene set analysis methods. An implementation of the proposed method is publicly available. We suggest using Silver to evaluate existing and new gene set analysis methods. Evaluation using Silver provides a better understanding of current methods and can aid in the development of gene set analysis methods to achieve higher specificity without sacrificing sensitivity.

## 1. Introduction

High-throughput technologies are widely used to monitor the expression activity of many genes in a single experiment. Analyzing high dimensional data resulting from these technologies is challenging. Gene set analysis, also known as enrichment analysis, is widely used to address this challenge and to gain insight from the resulting data [1].

Gene set analysis employs *a priori* knowledge of groups of genes that are known to be associated with biological components, processes, or functions. Such groups of genes, also referred to as gene sets or pathways, can be extracted from knowledgebases such as GO [2] and KEGG [3]. Hereafter, we refer to such a collection of gene sets as a gene set database. Given a gene set database and a case-control gene expression dataset, gene set analysis aims to find gene sets from the database that are differentially enriched when contrasting case and control samples—for example, a pathway that is activated (or deactivated) in case samples when compared to controls.

Many gene set analysis methods have been developed [4,5,6,7,8,9,10], and it has been shown that different gene set analysis methods may lead to significantly different results in terms of gene sets reported as differentially enriched [11,12]. Considering the large number of available methods, it is natural to wonder which method should be used. Answering this question in a quantitative manner requires a gold standard expression dataset where differentially enriched gene sets are known *a priori*. Due to the absence of such gold standard datasets, real datasets with presumed enrichment status of gene sets [13,14] and synthesized datasets [15,16,17] have been used. Unfortunately, these have had shortcomings, and evaluating gene set analysis methods remains a challenge.

Evaluations using real datasets are often based on questionable assumptions about the differential enrichment statuses of the gene sets. For example, Tarca et al. [13] used 42 microarray datasets from GEO. For each dataset of a specific disease, they assumed one KEGG or Metacore pathway/gene set associated with that disease is differentially enriched. When analyzing these datasets, they used the *p*-values associated with the 42 corresponding target gene sets to assess sensitivity. This approach ignores the enrichment status of the rest of the gene sets in the gene set database. In addition, this approach is reliant on the quality of the expression datasets used for evaluations and assumes that the pathways/gene sets associated with the diseases under study have been differentially enriched. Therefore, this approach cannot be relied on for a fine-grained evaluation of gene set analysis methods.

Evaluations using synthesized datasets have also been conducted relying on oversimplifying assumptions that may not represent the true nature of real data [15,16,17]. Such evaluations have often used normally distributed expression values with no gene–gene correlations [16,17] or with constant correlations [15], even though more complex gene–gene correlations are commonplace and profoundly impact the results of gene set analysis [18]. In fact, the complex correlation pattern of genes is essential for understanding the systems-level biology of an organism. Consequently, a wide variety of methods are focused on gene co-expression network analysis [19,20]. Moreover, normally distributed values with a constant mean and standard deviation ignores the heterogeneity of variance, which is a common phenomenon in high-throughput data [21,22]. Hence, the resulting datasets may be biased toward or against a specific method or class of methods. For example, Efron and Tibshirani [16] generated simulated expression values using independent standard normal distributions, where differential expression of a gene was simulated by adding a constant value to the gene expression values in case samples. As another example, Nam and Kim [17] simulated an expression profile using a standard normal distribution. To simulate differential expression, they added a random value between 0.5 and 1 to the expression measures in the case samples. In other research, Ackermann and Strimmer [15] generated expression datasets using a 600-dimensional multivariate normal distribution with variances of 1. Each profile contained 520 background (non-informative) genes with a mean gene expression value of zero and with no correlation between genes. The remaining 80 genes were assigned to four nonoverlapping gene sets each containing 20 genes. To investigate different hypotheses, they created gene sets with and without gene–gene correlation. They modeled the correlations between genes within a gene set using constant correlation values of 0.6 and −0.6. They also modeled the differential expression of genes by mean expression value differences of 1 and 0.75. Therefore, in the resulting expression profile, almost 87% of genes, i.e., 530/600, had a standard normal distribution with zero mean and no correlation. Consequently, even a small deviation from the background distribution, i.e., standard normal distribution, can be detected by gene set analysis methods that use gene-sampling or parametric methods for significance assessment. That is, generated datasets based on this approach are biased in favor of methods that use the aforementioned approaches for significance assessments of gene set scores.

Furthermore, previous evaluations have often utilized gene sets of equal size, as opposed to gene sets of varying sizes (the typical situation), which have been reported to affect some methods [23]. Another shortcoming of these evaluations is that they only consider non-overlapping gene sets. Therefore, overlapping of gene sets and its effect on the specificity of gene set analysis [24] have been overlooked.

Recently, Mathur et al. created artificial gene expression datasets in a more realistic manner [25]. They conducted a systematic comparison of 4 gene set analysis methods using real expression datasets by sampling with replacement from control and case samples. In each experiment, they selected two *a priori* known gene sets and randomly chose a proportion of their genes to be differentially expressed. They simulated differential expression “by shifting the gene expression in the [simulated] case groups according to a range of values” [25]. To calculate the power of a gene set analysis method, using a bootstrapping approach, they simulated 100 datasets by sampling with replacement. Control samples were selected from the control samples of the original datasets. Case samples were chosen from the case samples of the dataset containing differentially expressed genes. A gene set analysis method was run for each of these 100 datasets, and its power was reported as the proportion of datasets for which the target gene sets were predicted as significant, i.e., with an adjusted *p*-value less than 0.05. They also suggested three different scenarios for estimating the false positive rate for a gene set analysis method using: (1) a standard normally distributed expression dataset, (2) a dataset resulting from permuting sample labels of the original dataset, and (3) the normalized and centered version of the original dataset. The first approach suffers from the same shortcomings as other synthetic datasets, as explained earlier. The second and third approaches lead to a discrepancy between the distributions of the simulated and the original datasets. In a dataset simulated by the second approach, the simulated controls (and also cases) contained a heterogeneous mixture of both actual control and case samples. The third approach also leads to datasets with zero average expression values; therefore, the simulated datasets were not representative of the real data. The approach by Mathur et al. is also computationally demanding and almost impractical for evaluating computationally expensive methods such as SetRank [26]. Furthermore, to evaluate false positive rate, the approach by Mathur et al. only considers a few target gene sets and ignores the enrichment status of the rest of gene sets. Therefore, it cannot provide a precise evaluation of specificity of gene set analysis methods, which is known to be one of the main challenges for many gene set analysis methods [1].

Despite the existence of many studies comparing gene set analysis methods, there is no consensus regarding the method of choice for a given experiment, and existing guidelines and suggestions are often contradictory [27]. In this research, we propose Silver, a framework for evaluating gene set analysis methods. The framework synthesizes gene expression datasets without relying on oversimplifying assumptions, such as normally distributed expression values and zero or constant gene–gene correlations. The synthesized expression datasets preserve the true distribution of gene expression values and retain complex gene–gene correlation patterns. This approach incorporates gene set overlap, which has been shown to have a significant impact on the results of gene set analysis methods [24]. Additionally, it is computationally affordable as it does not rely on bootstrapping. After synthesizing the expression datasets, Silver follows a quantitative approach for comparing gene set analysis methods. In the following section, we describe the methodology for synthesizing expression datasets and the quantitative approach used by Silver for comparing gene set analysis methods.

We showcase the utility of Silver by providing a comprehensive evaluation of ten commonly used gene set analysis methods, including a recent method aimed at increasing specificity. We show that the expression datasets generated by Silver are more realistic and follow the same distribution as real data. Additionally, we demonstrate that the quantitative approach offered by Silver is capable of identifying the known limitations of current gene set analysis methods, which cannot be observed when using other methodologies when evaluating gene set analysis methods.

## 2. Materials and Methods

Silver, the proposed framework for evaluation of gene set analysis methods, is presented in this section. The framework consists of a methodology for synthesizing "almost gold" standard expression datasets and a quantitative approach for comparing gene set analysis methods. We have made Silver publicly available as a GitHub repository at https://github.com/FarhadMaleki/silver, accessed on 22 August 2021.

**Figure 1:**
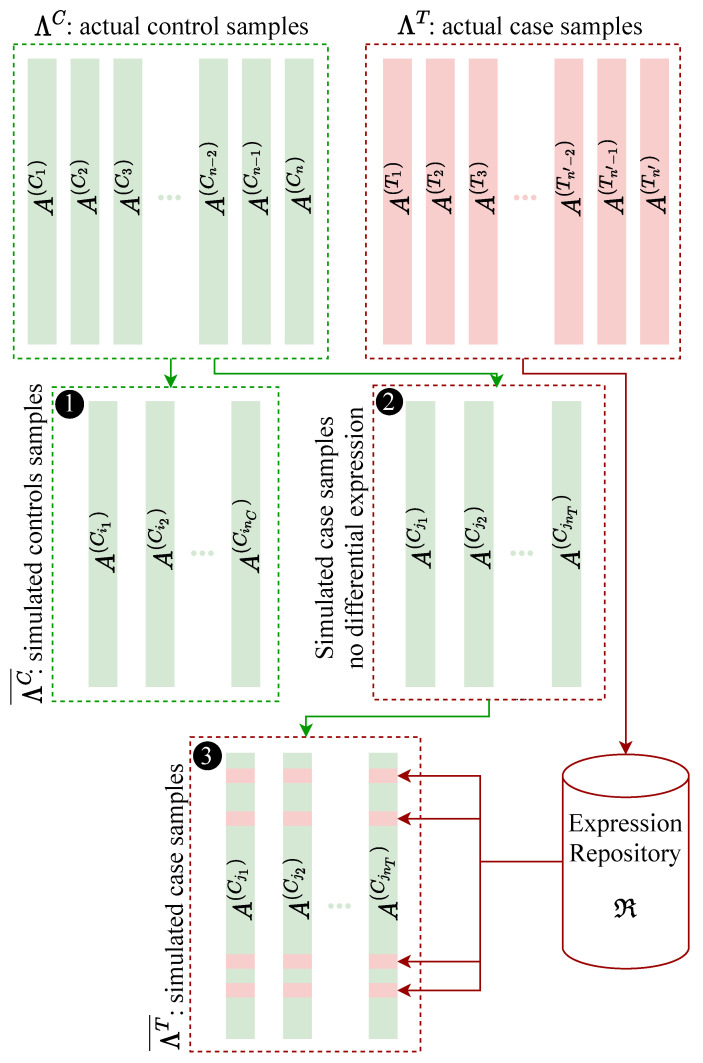
Visualization of the methodology used by Silver to synthesize expression datasets. The control samples ($\Lambda^{C}$), and the case samples ($\Lambda^{T}$) of the original dataset are shown in green and red, respectively. To simulate control samples, Silver uses a subset $\bar{\Lambda^{C}}=\left\{A^{\left(C_{i_{1}}\right)},\ldots ,A^{\left(C_{i_{n_{C}}}\right)}\right\}$ of original control samples shown in box 1. To simulate case samples, Silver also uses another subset $\bar{\Lambda^{T}}=\left\{A^{\left(C_{j_{1}}\right)},\ldots ,A^{\left(C_{j_{n_{T}}}\right)}\right\}$ of control samples shown in box 2. Silver creates a repository of real expression values *ℜ* by sampling from the original cases from $\Lambda^{T}$. The repository *ℜ* can be considered as a table of expression measures with $n_{T}$ columns, where $n_{T}$ is the desired number of cases to be simulated. Each row of *ℜ* contains $n_{T}$ measures sampled from one row of the actual cases $\Lambda^{T}$. For a given gene, Silver simulates differential expression by finding expression measures that satisfy a criterion for differential expression of that gene and replaces the expression measures in the simulated cases (from box 2) for that gene in order to create new case samples shown in box 3. Criteria implemented for differential expression include *t*-test, Wilcoxon rank-sum test, and simple fold-change.

Silver uses actual expression datasets to simulate a case-control dataset where the expression status of genes within a gene set is known *a priori*. Silver uses a subset of control samples as the simulated control samples. It also utilizes a subset of control samples from the actual dataset to generate the simulated case samples. It is expected that these selected samples contain no statistically significant differential expression; therefore, Silver introduces differential expression for a group of genes. This group of genes could be from one or several gene sets. The list of genes, and their magnitudes of differential expression, are considered as input to the algorithm. To avoid oversimplifying assumptions regarding the distribution of expression values for these genes, Silver utilizes the expression measures from the actual case samples. Figure 1 illustrates the process used for simulating a case-control dataset. The remainder of this section explains the methodology used for synthesizing an expression dataset in detail.

### 2.1. Synthesizing Expression Datasets

This section provides a mathematical description of the methodology used by Silver for synthesizing expression datasets (refer to Figure 1 for a visual overview). To synthesize an expression profile with $n_{C}$ controls and $n_{T}$ cases, first we identify an actual expression dataset $\Lambda =(\Lambda^{C},\Lambda^{T})$ where $\Lambda^{C}=\left\{A^{\left(C_{1}\right)},\ldots ,A^{\left(C_{n}\right)}\right\}$ are *n* control samples and $\Lambda^{T}=\left\{A^{\left(T_{1}\right)},\ldots ,A^{\left(T_{n^{\prime }}\right)}\right\}$ are $n^{\prime }$ case samples. Each $A^{C_{i}}$ and $A^{T_{j}}$ is a vector of the expression levels for *m* genes. It is required that $n\geq n_{C}+n_{T}$ and $n^{\prime }\geq n_{T}$.

Then, $\bar{\Lambda^{C}}=\left\{A^{\left(C_{i_{1}}\right)},\ldots ,A^{\left(C_{i_{n_{C}}}\right)}\right\}$ and $\bar{\Lambda^{T}}=\left\{A^{\left(C_{j_{1}}\right)},\ldots ,A^{\left(C_{j_{n_{T}}}\right)}\right\}$ are created through random sampling without replacement so that $\bar{\Lambda^{C}}$ and $\bar{\Lambda^{T}}$ are disjoint subsets of $\Lambda^{C}$. $\bar{\Lambda^{C}}$ and $\bar{\Lambda^{T}}$ together form an expression matrix, where each column corresponds to a member of $\bar{\Lambda^{C}}$ or $\bar{\Lambda^{T}}$ and each row corresponds to the expression values for a gene $g_{k}$ (1≤k≤m) across samples in $\bar{\Lambda^{C}}$ and $\bar{\Lambda^{T}}$. In other words, the generated expression matrix contains $n_{C}+n_{T}$ columns and *m* rows, where *m* is the number of genes in the original dataset.

Given a set $L\subset \{g_{1},\ldots ,g_{m}\}$—where *L* is a user input representing the set of genes to be differentially expressed—for each gene $g_{t}$ in *L* we adjust the expression levels of $g_{t}$ in $\bar{\Lambda^{T}}$. This is accomplished by simulating differential expression through the following process. We first create *ℜ*, which is a table of expression values with *m* rows and $n_{T}$ columns; each column is selected from the actual cases $\Lambda^{T}$ through random sampling without replacement. The columns of *ℜ* are $A^{\left(T_{\ell_{1}}\right)},\ldots ,A^{\left(T_{\ell_{n_{T}}}\right)}$, where $1\leq \ell_{1}<\cdots <\ell_{n_{T}}\leq n^{\prime }$. Each row of *ℜ* represents the expression values for a gene across $A^{\left(T_{\ell_{1}}\right)},\ldots ,A^{\left(T_{\ell_{n_{T}}}\right)}$. This table is used to simulate differential expression of each gene in *L* according to some specified criterion. Among criteria one can use are *t*-test, Wilcoxon rank-sum test, and median fold change. To simulate differential expression of a gene $g_{t}$ in *L* by a given fold change $FC(g_{t})$, we randomly choose a row *e* from rows of *ℜ* that satisfies the differential expression criterion considering the simulated control expression values for $g_{t}$ (from $\bar{\Lambda^{C}}$) and $FC(g_{t})$. The current expression values for gene $g_{t}$ in $\bar{\Lambda^{T}}$ (a vector of size $n_{T}$) are replaced with the vector of expression measures from row *e* of *ℜ* (row *e* of *ℜ* does not necessarily correspond to gene $g_{t}$). The choice of genes selected for differential expression (*L*) depends on the purpose of simulation and is an input.

Note that given the initial expression level of $g_{t}$, if the intended fold change value—which is a user input—is unrealistically high or low considering the distribution of expression values in the original dataset, a row *e* that meets the criterion might not exist. In such rare cases, optionally, expression values for $g_{t}$ can be chosen based on a normal distribution. The mean of the normal distribution is determined to meet the fold-change value requirement for differential expression (or another criterion of choice) and the standard deviation is a user-defined constant. After updating expression values for genes in *L*, $\left(\bar{\Lambda^{C}},\bar{\Lambda^{T}}\right)$ is returned as the synthesized dataset.

As the proposed method inherits the characteristics of an input dataset, care should be taken when choosing input datasets. Having a MDS (multidimensional scaling) plot of the input datasets where controls and cases cluster separately might be a good rule of thumb for choosing a dataset of sufficient quality [11].

The choice of gene set database has been shown to be another important factor in gene set analysis [28]. Instead of following the common approach of generating a small number of non-overlapping artificial gene sets of equal size, because of the way we synthesize the gene expression datasets, we are able to use real gene set databases to evaluate gene set analysis methods. This is possible as we synthesize expression profiles using real data and therefore retain real gene identifiers along with their gene expression characteristics from an actual dataset.

Due to the availability of large-scale expression datasets that can be used for evaluation of gene set analysis methods, by default, Silver uses a sampling without replacement approach. However, for some applications, this choice might be limiting due to the requirements regarding sample sizes. Therefore, we also provide the use of sampling with replacement as an option. This alleviates the need for a large real dataset by relieving the following conditions (which were introduced earlier in this section): $n\geq n_{C}+n_{T}$ and $n^{\prime }\geq n_{T}$.

### 2.2. Quantitative Approach

We utilized the aforementioned procedure to synthesize expression datasets where the enrichment status of given gene sets is known apriori. To achieve this goal, we selected a group of gene sets $G_{1},\ldots ,G_{q}$—from a gene set database G—and synthesized an expression dataset, with genes in these gene sets being differentially expressed. However, not only do we need to consider $G_{1},\ldots ,G_{q}$ as being differentially enriched, but to reflect actual data we also need to consider gene sets that “substantially overlap” with $G_{1},\ldots ,G_{q}$ as being differentially enriched. However, there is no consensus about what should be considered as a “substantial overlap”. We used a methodology similar to that proposed by Maleki and Kusalik [24] to address this ambiguity and to determine the enrichment status of gene sets.

**Figure 2:**
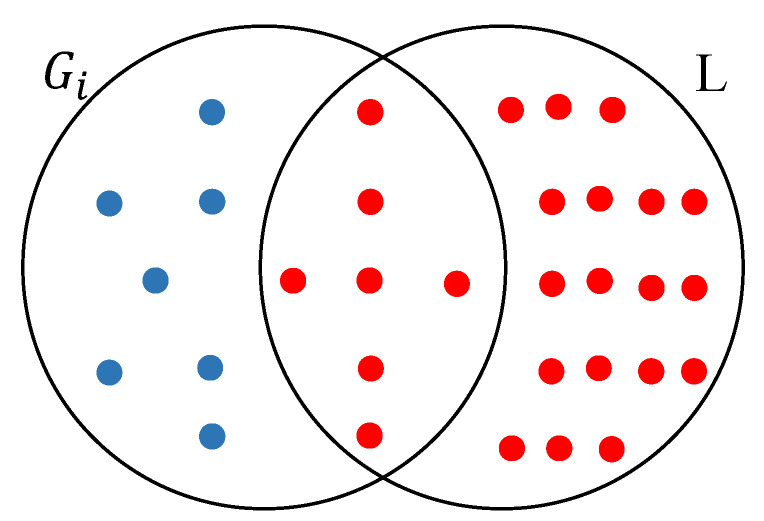
An example of coverage score. Differentially expressed genes are shown as red circles. $f\left(G_{i},L\right)=\frac{1}{2}$; i.e., 50% of genes in $G_{i}$ are differentially expressed.

Assuming that *L* is the list of all genes that are differentially expressed in the synthetic dataset $\left(\bar{\Lambda^{C}},\bar{\Lambda^{T}}\right)$, we consider a gene set $G_{i}$ in G as truly differentially enriched if the following inequality holds:

(1) $$f\left(G_{i},L\right)=\frac{∥G_{i}\cap L∥}{∥G_{i}∥}>\gamma$$

where γ is a value between 0 and 1 and ∥•∥ is set cardinality. $f(G_{i},L)$ represents the proportion of genes in $G_{i}$ that are differentially expressed. Hereafter, we refer to *f* as the coverage score of $G_{i}$ given *L* or simply as the coverage score of $G_{i}$ in situations where *L* can be inferred from the context. Figure 2 illustrates an example of calculating coverage score.

Since there is no consensus in the research community about an appropriate value of γ, we used a wide range of values for γ from 0.1 to 0.99 and evaluated a gene set analysis method for each value. We present results for γ values equal to 0.1, 0.3, 0.5, 0.9, and 0.99; and the results for other values are available from the authors upon request.

Knowing the truly enriched gene sets in a simulated dataset and results of a gene set analysis method for that dataset, we can then quantitatively evaluate the result of a gene set analysis method in terms of sensitivity and specificity. A reliable gene set analysis method should achieve both high sensitivity and high specificity.

### 2.3. Evaluation Using Silver

Using the proposed framework, here we evaluate ten commonly used gene set analysis methods: PAGE [6], GSEA (both gene permutation and phenotype permutation versions) [8], PLAGE [9], GAGE [7], ssGSEA [4], ROAST [10], GSVA [5], over-representation analysis (ORA) [29], and SetRank [26], a more recent method claiming to increase specificity. We used the following R packages in this study: *GSVA* package version 1.18.0 for GSVA, PLAGE, and ssGSEA; the *limma* package version 3.34.9 for ROAST; the *gage* package version 2.20.1 for PAGE and GAGE; and *SetRank* version 1.1.0 for SetRank. ORA was run using the WebGestalt online service [30]. GSEA was obtained from the Java-based application v3.0 (build 0160) at the Broad Institute software page for GSEA (http://software.broadinstitute.org/gsea/downloads.jsp, accessed on 22 August 2021).

The gene set analysis methods are evaluated using data simulated from two microarray datasets and 1 RNA-seq dataset, downloaded from GEO and each used as an original dataset Λ. The microarray experiments were case-control experiments in humans from the Affymetrix GeneChip Human Genome U133 Plus 2.0 microarray platform from studies of renal cell carcinoma tissue (77 controls and 77 cases, GSE53757) and skin tissue in psoriasis patients (64 controls and 58 cases, GSE13355). These datasets were normalized, as described in a previous work, and resulted in each microarray dataset containing 20,514 genes [31].

The RNA-seq dataset originated from normal and lesional psoriatic skin (82 controls and 92 cases, GSE54456). The 80-base single-stranded reads were trimmed with Trimmomatic 0.36 and mapped to the GRCh38 human genome using STAR 2.2.51 to obtain raw counts. The dataset was normalized using TMM normalization from the *edgeR* R package. The Ensembl gene IDs were translated to human Entrez gene IDs using *biomaRt*. Ensembl IDs (and also Probe IDs for microarrays) were collapsed to obtain a unique set of Entrez gene identifiers using methods described in a previous work [31]. This resulted in the RNA-seq dataset containing 16,826 genes.

GO gene sets (a total of 5917) were extracted from MSigDB version 6.1 and used as our gene set database G. For each expression dataset, genes not represented in the dataset were removed from these gene sets.

From each original dataset, we simulated a dataset containing 20 controls and 20 cases. Ten gene sets—($G_{1}$) GO:0003823, ($G_{2}$) GO:0019724, ($G_{3}$) GO:0060070, ($G_{4}$) GO:0005126, ($G_{5}$) GO:0008009, ($G_{6}$) GO:0030851, ($G_{7}$) GO:0002544, ($G_{8}$) GO:0045087, ($G_{9}$) GO:0002253, and ($G_{10}$) GO:0006954—of various sizes (see Table 1) associated with immune system processes were selected for being differentially enriched in each simulated dataset. The list *L* contains 1106 unique genes from the ten gene sets. Genes in *L* are differentially expressed with mixed proportions of up- and down-regulated genes (see Table 1) and absolute log2 fold change values between 1 and 3. Hereafter, we refer to these ten gene sets as the target gene sets. Additionally, independent two-sample *t*-test is used as the differential expression criterion.

## 3. Results

**Figure 3:**
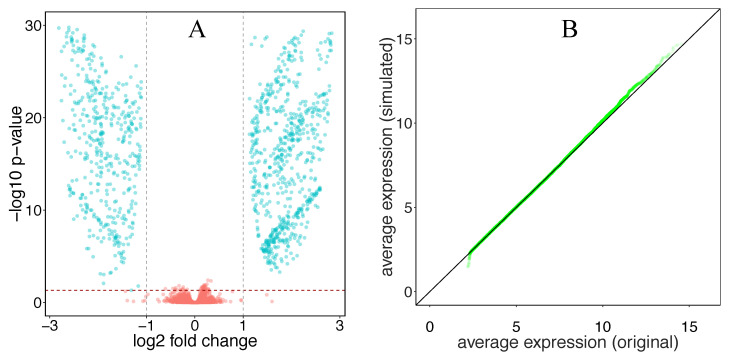
(**A**) The volcano plot shows differentially expressed genes resulting from simulated data (20 control and 20 case samples) using dataset GSE53757. The blue points represent genes that were differentially expressed and the red points represent non-differentially expressed genes. The vertical dotted lines indicate the log fold change thresholds that were considered significant. The red horizontal line indicates the *p*-value cutoff, 0.05. The *p*-values were obtained by performing differential expression analysis using the *limma* R package. (**B**) A Q–Q plot of the average expression values of cases versus controls for the synthesized dataset generated from GSE53757.

Figure 3 illustrates the volcano plot and also a Q–Q plot of the average expression value of cases versus controls for the synthesized dataset generated from GSE53757. This volcano plot, and the volcano plots in Figure S1 in Supplementary Materials: File 1, resembles a typical volcano plot resulting from differential expression analysis of real data. Further, a two-sample Kolmogorov–Smirnov test was used to assess if the average expression levels in a simulated dataset follow the same distribution as the real dataset it was generated from. As indicated in Table 2, the null hypothesis cannot be rejected for any of the datasets, suggesting that the distributions are the same.

We also compared several simulated datasets used for the evaluation of gene set analysis methods [15,16,17]. Since these synthesized datasets use different inputs and different synthetic gene set databases, it is not possible to directly compare them. Instead, we compared several methods for simulating datasets to Silver by their ability to shed light on the lack of specificity of PAGE, a gene set analysis method from a class of methods that have been reported in the literature as non-specific when applied to real datasets [7,18,32]. Using several synthesized datasets used for the evaluation of gene set analysis methods, we observed that these datasets could not reveal the lack of specificity of PAGE. On the contrary, they reported PAGE as a reliable procedure (results provided in Supplementary Materials: File 2). Silver, however, was able to demonstrate the lack of specificity of PAGE.

**Figure 4:**
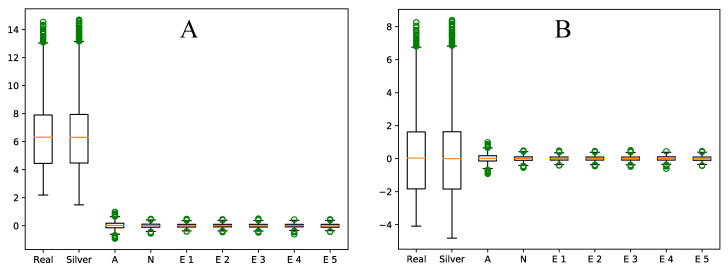
Distributions of the expression values of different synthesized datasets (**A**), including a dataset synthesized with Silver, compared to the distribution of expression values from a real dataset (GSE53757). The datasets labeled “E 1” to “E 5” were introduced by Efron and Tibshirani [16]. The dataset labeled “N” was introduced by Nam and Kim [17], and the dataset labeled “A” was introduced by Ackermann and Strimmer [15]. While the dataset generated by Silver closely mirrors the real dataset (GSE53757), the other simulated datasets show substantial differences from the real data. Additionally, this difference is not due to a constant shift in average expression values, as illustrated by the box plot representing the distribution of centered average expression values for all datasets (**B**).

We also showed that the synthesized data using Silver follow the same distribution as real expression data (see Figure 3 and Table 2). This was expected, as Silver utilizes real data for selecting control samples, and almost all expression measures used for differential expression of genes in Silver come from real expression measures. As illustrated in Figure 4A, the other simulated datasets, unlike the datasets synthesized by Silver, showed substantial differences from real datasets. To make sure that these differences are not only due to a constant difference in expression values, we compared the centered average expression values for all datasets. As depicted in Figure 4B, there were substantial differences between the distributions of expression values of other datasets and real data, even after centering. The same pattern was observed for the other datasets used in this study (see Figures S6 and S7 in Supplementary Materials: File 1). To statistically verify these observations, we used two-sample Kolmogorov–Smirnov tests to assess if there is a statistical difference between the distribution of expression values of a synthesized dataset (after centering) with that of a real dataset. Table 3 shows the results of these tests. The results indicated that there is no statistically significant differences between the average expression values in the dataset simulated by Silver and those of real expression datasets, whereas the average expression values of the other simulated datasets significantly differ from those of real datasets. The same pattern was observed for the other datasets used in this study (see Tables S3 and S4 from Supplementary Materials: File 1).

We now demonstrate the utility of Silver as a means to evaluate the ten gene set analysis methods. For each method, the default parameters—as suggested by its author(s)—were used. To achieve comparable results, the Benjamini–Hochberg adjustment [33] for multiple comparison with a false discovery rate of 0.05 was applied to the reported *p*-values for each method.

**Figure 5:**
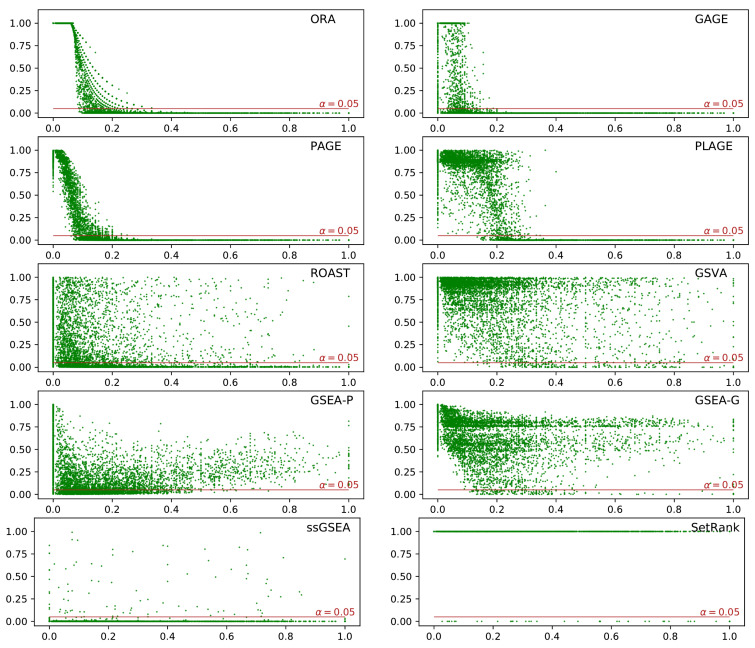
Scatter plots of the relationship between gene set coverage (x-axis) and the statistical significance (adjusted *p*-value) of the results of each method (y-axis). Each point in green represents a gene set. The red line shows a *p*-value cutoff of α=0.05. Since SetRank only returned statistically significant results (points under the red line), we assigned a *p*-value of 1 to visualize the coverage scores for non-significant results. Note that no cut-off value of γ was applied in any of these scatter plots.

Each plot in Figure 5 illustrates the reported *p*-values—resulting from running a method—for each gene set in the database versus its coverage score given the list of differentially expressed genes for the dataset synthesized from GSE53757. These plots show the lack of specificity of the methods under study. Almost all methods reported a large number of gene sets as being differentially enriched regardless of the coverage scores. As depicted in Figure 5, ORA, GAGE, PAGE, and PLAGE reported gene sets with high coverage—i.e., gene sets with a large proportion of their genes being differentially expressed—as being differentially enriched. Unexpectedly, the other methods reported some of the gene sets with high coverage as non-enriched.

**Figure 6:**
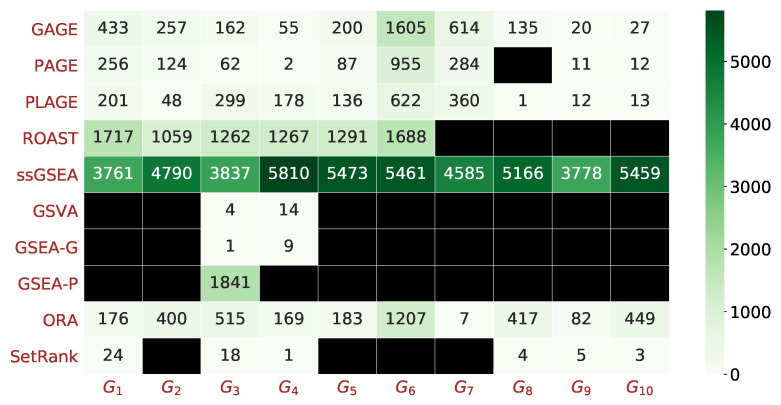
Heat map of the rank of the 10 target gene sets as reported by each method. The results of each method were sorted based on the adjusted *p*-values (smallest to largest); the rank of each target gene set was determined as its rank in the sorted list. The rank was then recorded in each cell and encoded by the color of the cell, where a darker green indicates a later position in the sorted list. A black cell with no number shows that the adjusted *p*-value was not less than α=0.05.

Figure 6 shows the ranks of the target gene sets based on adjusted *p*-values reported by each method. The heat map shows that the rankings of the target gene sets substantially differ across methods, with some methods not being able to report some of the target gene sets as differentially enriched. Additionally, GSEA-G and GSVA only ranked gene sets highly when most of their genes were up-regulated. GSEA-P and ssGSEA reported the majority of target gene sets near the bottom of their results. GAGE, PAGE, PLAGE, and ORA ranked the target gene sets higher in comparison to other methods. SetRank, while ranking six of the ten target gene sets highly, failed to report the other four target gene sets.

Table 4 shows the sensitivity and specificity of the methods under study when analyzing the dataset synthesized from GSE53757 across γ values. As depicted in Table 4, GSEA-G, GSVA, and SetRank achieved high specificity with the consequence of having low sensitivity. GAGE, PAGE, and ssGSEA achieve high sensitivity while sacrificing specificity, with ssGSEA being the least specific. These results are consistent across all γ values. PLAGE, while achieving high sensitivity (across γ>0.1), also achieved 0.7 or higher specificity. However, due to the sheer size of gene set databases (5000+ for GO gene sets, and 16,000+ for MSigDB), such specificity is not high in absolute terms and leads to hundreds to thousands of false positives.

**Figure 7:**
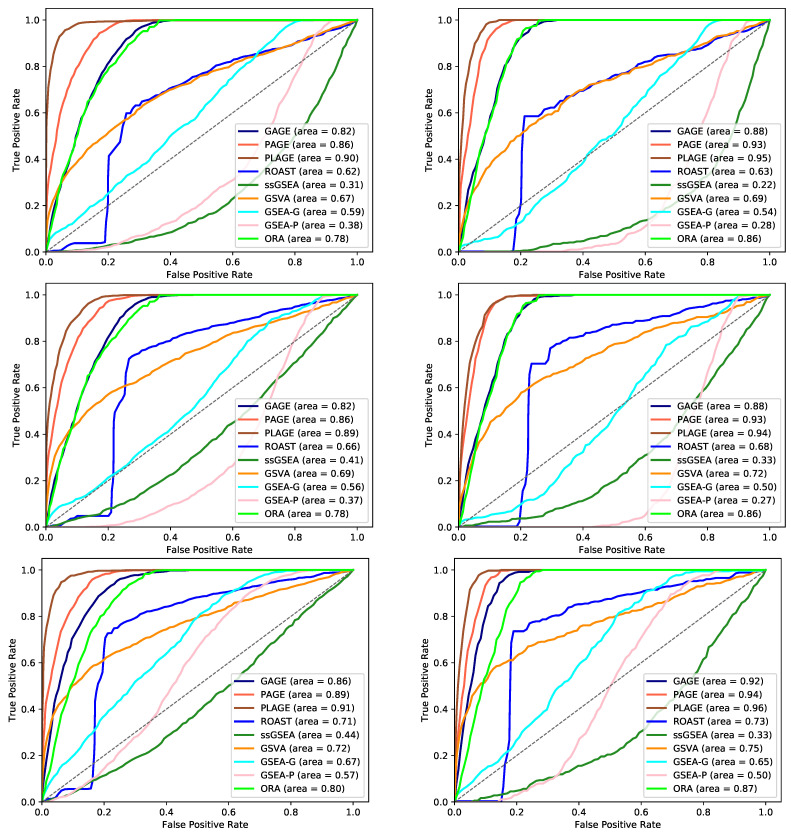
Receiver operator characteristic curves (ROC) for each method using γ=0.3 (**left column**) and 0.5 (**right column**) for the datasets synthesized based on microarray datasets, GSE53757 and GSE13355; and RNA-Seq dataset GSE54456 (from top to bottom). The plots show the relationship between the true positive rate (y-axis) and the false positive rate (x-axis). A method with higher area under the curve (shown for each method) is considered better. The black dotted diagonal line (y = x) represents a method with random ordering of significance values. Note that SetRank is not included in the ROC curves as the order of the non-significant differentially enriched gene sets is not reported by this method.

Figure 7 shows the receiver operator characteristic (ROC) curves for the results of each method for all three synthetic datasets using two values of γ. The ROC curves, and Table 4, suggest that GSEA (both gene permutation and phenotype permutation versions) and ssGSEA performed poorly regardless of the value of γ. Additionally, GSVA performed moderately better than these methods. ORA, ROAST, PAGE, GAGE, and PLAGE achieved a relatively higher area under the curve. This supports the reliability of the most statistically significant results reported by these methods.

Results using the other two simulated datasets were consistent with the observations reported in Figure 5 and Figure 6 and Table 4 (see Tables S1 and S2 and Figures S2–S5 in Supplementary Materials: File 1).

## 4. Discussion

We proposed Silver, a framework for evaluating gene set analysis methods consisting of a method for synthesizing expression datasets and a quantitative approach for evaluating gene set analysis methods. While the proposed methodology does not generate gold standard datasets, it is capable of generating expression datasets without relying on common oversimplifying assumptions and preserves the characteristics of real (input) datasets. The synthesized datasets inherit the distribution of expression values and complex gene–gene correlations from real data, preserving technical and biological variability. This was expected, as the proposed method incorporates real data, and was confirmed by Kolmogorov–Smirnov tests shown in Table 2 and Table 3 and the visualizations in Figure 3 and Figure 4; and Figures S1, S6, and S7. Our observations were consistent across both RNA-seq and microarray datasets. This means the methodology is also not limited to a specific gene expression dataset or platform. Although we used the methodology for simulating expression datasets as part of an evaluation of gene set analysis methods, its utility is not limited to this role, and it can be used in any context where one needs expression datasets with control over differentially expressed genes. Moreover, Silver utilizes real gene set databases to avoid using artificial databases of non-overlapping gene sets of equal size that are unrealistic and substantially affect the results of gene set analysis methods [24].

We evaluated a comprehensive list of gene set analysis methods, providing key insights into weaknesses and strengths of these methods. A compelling observation revealed by Figure 5 and Figure 6 (and Figures S3–S5 in Supplementary Materials: File 1) is that some methods—such as ROAST, GSVA, SetRank, GSEA-G, and GSEA-P—did not report certain gene sets as being differentially enriched even when all the genes in those gene sets were differentially expressed. For example, gene set GO:0002544—with 15 genes of which 7 were up-regulated and 8 were down-regulated—was not reported as differentially enriched by the aforementioned methods. This suggests an inadequacy of these methods in detecting gene sets with both up- and down-regulated genes, which would lead to under-reporting of pathways in which, by definition, some genes must be up-regulated and some down-regulated during a biological process or function.

Using Silver, ORA, GAGE, PAGE, and PLAGE achieved high sensitivity by predicting all gene sets with high coverage as being differentially enriched, as depicted in Figure 5 and Figure 7; and Figures S2 and S3 in Supplementary Materials: File 1. However, these methods also predicted a large number of the gene sets with low coverage as being differentially enriched. The gene sets with low coverage often are not biologically informative, which increases the difficulty of interpreting the results of these methods.

Given a gene set $G_{i}$ and a list of differentially expressed genes *L*, the significance assessment for differential enrichment of $G_{i}$ is a function of the number of differentially expressed genes that occur in $G_{i}$; i.e., $∥L\cap G_{i}∥$, which is the numerator in Equation (1). Therefore, as expected, ORA predicted all gene sets with high coverage as differentially enriched.

ORA also predicted some gene sets with low coverage values (for example 0.1) as differentially enriched. For instance, considering 1106 differentially expressed genes out of the total of 20,514 genes on the microarrays, a gene set with 200 genes of which only 20 genes were differentially expressed (a coverage score of 0.1) led to a *p*-value of 0.0058. Gene sets like this were reported as differentially enriched even after correction for multiple comparisons. Gene sets with low coverage tend to be large gene sets. For instance, among the 1060 gene sets with coverage scores less than 0.12, 168 had sizes greater than or equal to 200, and the remaining 982 gene sets had sizes less than 200. Of these 1060 gene sets, 58 gene sets were predicted by ORA as being differentially enriched, all with sizes greater than or equal to 200. This shows that for low coverage gene sets, ORA is biased toward large gene sets, which are often biologically irrelevant or less informative.

PAGE uses a one sample *z*-test to examine if there is a significant difference between the average gene expression fold change of genes in $G_{i}$ compared to that of genes not in $G_{i}$. Therefore, the results of PAGE are also a function of the number of differentially expressed genes in $G_{i}$. In this particular experiment, the results are a function of the number of genes in $G_{i}$ with an absolute fold change of two or higher. This explains the comparable results for PAGE and ORA. From Table 4, PAGE also suffers from a lack of specificity. Lack of specificity of PAGE can be attributed to the calculation of the z-statistic for each gene set. The z-statistic is calculated based on the fold change for each gene in a gene set between the average expression values of case samples and control samples for that gene. The z-statistic may significantly change even with the differential expression of a small percentage of genes in $G_{i}$.

GAGE is another parametric method. It uses a two-sample *t*-test to examine if there is a significant difference between the average gene expression fold change of genes in $G_{i}$ compared to that of genes not in $G_{i}$. However, GAGE uses one-on-one comparisons between control and case samples to calculate the fold change values. Therefore, it is more sensitive to changes in expression values leading to predictions of gene sets as differentially enriched with very low coverage values (for example 0.04), which are often biologically irrelevant.

PLAGE uses singular value decomposition to summarize the activity level of $G_{i}$ as a singular vector by capturing variability in expression values of genes in $G_{i}$. PLAGE uses this singular vector, referred to as a meta-gene, to assess if there is a significant difference between expression levels of genes in $G_{i}$ in control samples versus case samples. However, this meta-gene, as a characteristic of singular value decomposition, tends to capture the maximum variability of expression values. Therefore, the differential expression of a small percentage of genes in $G_{i}$ can substantially affect the meta-gene and lead to the prediction of $G_{i}$ as being differentially enriched.

The experiments also showed that ssGSEA suffers from a lack of specificity, and performed more poorly in these scenarios than random guessing. However, it should be mentioned that the available R package implementing this method [5] was not from the authors of ssGSEA. We strongly recommend against using the current implementation with default parameters.

SetRank was designed with the goal of increasing specificity. The available implementation does not report the non-significant gene sets; as such, a fair comparison of it with other methods via ROC curves is not possible. However, its scatter plot in Figure 5 and its sensitivity and specificity in Table 4 reveal a lack of sensitivity. As illustrated by Figure 6, four out of the ten target gene sets were not detected by SetRank. This might have been due to SetRank explicitly removing some gene sets based on a level of overlap between gene sets. However, excluding small gene sets where most or all genes are differentially expressed suggests that SetRank sacrifices sensitivity in favor of increasing specificity.

The proposed method has been designed to utilize real expression data to synthesize new expression datasets that inherit the characteristics of real expression data. Therefore, characteristics such as the quality of the input expression data might affect the synthesized datasets and potentially introduce bias in the simulation process and any downstream analysis. Therefore, we recommend avoiding evaluation and making conclusions using only a single input dataset.

In this study, we showcased the utility of Silver using a scenario of differentially enriching ten gene sets from processes related to immune system. However, this by no means is a comprehensive evaluation of these methods. We suggest studying various scenarios using gene sets from different phenotypes, only up-regulated (or down-regulated) gene sets of various sizes, and different levels of fold change gene expression values. In addition, we only used microarray and RNA-seq data in this work. We suggest synthesizing data from different gene expression modalities, such as single-cell data, for evaluating the performance of gene set analysis methods.

For all evaluations, we synthesized 20 control and 20 case samples, as it has been shown that gene set analysis results tend to be reproducible with at least 20 controls and 20 cases [32]. Silver provides a systematic means for the evaluation of gene set analysis methods and can be used for a comprehensive study of the performance of gene set analysis methods under different conditions, including various sample sizes. We suggest evaluating the performances of gene set analysis methods with different sample sizes and with unequal numbers of cases and controls as future research. Such studies are easily facilitated by Silver.

## 5. Conclusions

In this paper, we proposed Silver, a framework for generating synthetic data that avoids common oversimplifying assumptions. We showed the utility of this framework by evaluating a comprehensive list of gene set analysis methods. The evaluation revealed key insights about these methods. It showed a lack of specificity as the main challenge facing these gene set analysis methods. Moreover, we found that some methods lack sensitivity when dealing with gene sets/pathways that are mixtures of up- and down-regulated genes.

Considering the key insights revealed using Silver, we strongly discourage using artificial datasets that rely on oversimplifying assumptions, such as normally distributed expression values or non-overlapping gene sets of the same size, as they are not realistic and do not provide accurate evaluations of gene set analysis methods. We anticipate that using Silver as a means for evaluation of existing and new gene set analysis methods will provide a better understanding of these methods and lead to development of gene set analysis methods that achieve high specificity without sacrificing sensitivity.

## Figures and Tables

**Table 1:** Information about the target gene sets in this study, including the total number of genes in each set and the numbers of genes up-regulated or down-regulated in each target gene set.

Gene Set ID	Description	Down-Regulated	Up-Regulated	Gene Set Size
GO:0003823	Antigen binding	13	24	76
GO:0019724	B cell mediated immunity	21	27	67
GO:0060070	Canonical Wnt signaling pathway	2	65	92
GO:0005126	Cytokine receptor binding	44	206	250
GO:0008009	Chemokine activity	17	24	41
GO:0030851	Granulocyte differentiation	5	3	15
GO:0002544	Chronic inflammatory response	8	7	15
GO:0045087	Innate immune response	229	245	538
GO:0002253	Activation of immune response	142	155	383
GO:0006954	Inflammatory response	210	218	428

**Table 2:** The results of Kolmogorov–Smirnov tests comparing each original gene expression dataset to its corresponding dataset synthesized using Silver. The test results suggest that the expression values in each synthesized dataset follow the same distribution as its corresponding real dataset.

Dataset	Statistic	*p*-Value
GSE53757	0.009	0.427
GSE13355	0.007	0.674
GSE54456	0.012	0.212

**Table 3:** Comparison of the distribution of average expression values of a dataset synthesized by Silver and that of other simulated datasets used for the evaluation of gene set analysis methods. Datasets labeled “E 1” to “E 5” have been introduced by Efron and Tibshirani [16], the dataset labeled “N” has been introduced by Nam and Kim [17], and the dataset labeled “A” has been introduced by Ackermann and Strimmer [15]. To make sure that the differences between the distributions of average expression values are not due to a constant shift in expression values, all datasets were centered prior to conducting two-sample Kolmogorov–Smirnov tests. As the results of Kolmogorov–Smirnov tests indicate, the distribution of average expression of datasets simulated by Silver shows no statistically significant difference from that of real data (GSE53757). However, there are significant differences between the average expression values of the other datasets and those of the real data.

Dataset	Statistic	*p*-Value
Silver	0.009	0.427
E 1	0.448	<1.0 × 10${}^{-87}$
E 2	0.448	<1.0 × 10${}^{-87}$
E 3	0.443	<1.0 × 10${}^{-87}$
E 4	0.448	<1.0 × 10${}^{-87}$
E 5	0.449	<1.0 × 10${}^{-87}$
A	0.405	<1.0 × 10${}^{-87}$
N	0.445	<1.0 × 10${}^{-87}$

**Table 4:** The sensitivity (TPR) and specificity (TNR) of gene set analysis methods for data simulated from GSE53757.

	γ=0.1		γ=0.3		γ=0.5		γ=0.9		γ=0.99	
Method	TNR	TPR	TNR	TPR	TNR	TPR	TNR	TPR	TNR	TPR
GAGE	0.73	0.98	0.40	1.00	0.35	1.00	0.32	1.00	0.32	1.00
GSEA-G	1.00	0.05	0.98	0.07	0.97	0.03	0.97	0.14	0.97	0.16
GSEA-P	0.68	0.39	0.59	0.13	0.61	0.01	0.64	0.00	0.64	0.00
GSVA	1.00	0.07	0.99	0.16	0.98	0.19	0.96	0.42	0.96	0.44
PAGE	0.97	0.90	0.59	1.00	0.52	1.00	0.48	1.00	0.47	1.00
PLAGE	1.00	0.49	0.89	0.99	0.78	1.00	0.72	1.00	0.72	1.00
Roast	0.76	0.67	0.57	0.72	0.53	0.74	0.51	0.92	0.51	0.92
ssGSEA	0.01	0.98	0.01	0.96	0.01	0.94	0.02	0.97	0.02	0.96
SetRank	1.00	0.01	1.00	0.03	1.00	0.05	0.99	0.22	0.99	0.28
ORA	0.99	0.69	0.60	1.00	0.50	1.00	0.44	1.00	0.44	1.00

## Data Availability

The gene expression datasets used in this research are publicly available from Gene Expression Omnibus through record IDs of GSE53757, GSE54456, and GSE13355.

## Supplementary Materials

The following are available online at https://www.mdpi.com/article/10.3390/genes12101523/s1. Additional File 1: Supplementary Figures and Tables: This file includes the results of the analysis for the datasets and methods not presented in the main body of the paper. Additional File 2: Supplementary Excel file: This file includes the results of PAGE when different simulated datasets have been used for its evaluation.

## Abbreviations

The following abbreviations are used in this manuscript:

ORA	Over-representation analysis
GEO	Gene Expression Omnibus
GSEA-P	GSEA phenotype permutation
GSEA-G	GSEA gene permutation
