# Supplementary material for: Silver: Forging almost Gold Standard Datasets
Source: Genes (Basel). 2021 Sep 28;12(10):1523. doi: 10.3390/genes12101523 (PMC8535810; doi:10.3390/genes12101523)
Supplement: Supplementary file 1 [file genes-12-01523-s001.zip › File 1.pdf]

# Supplementary Materials

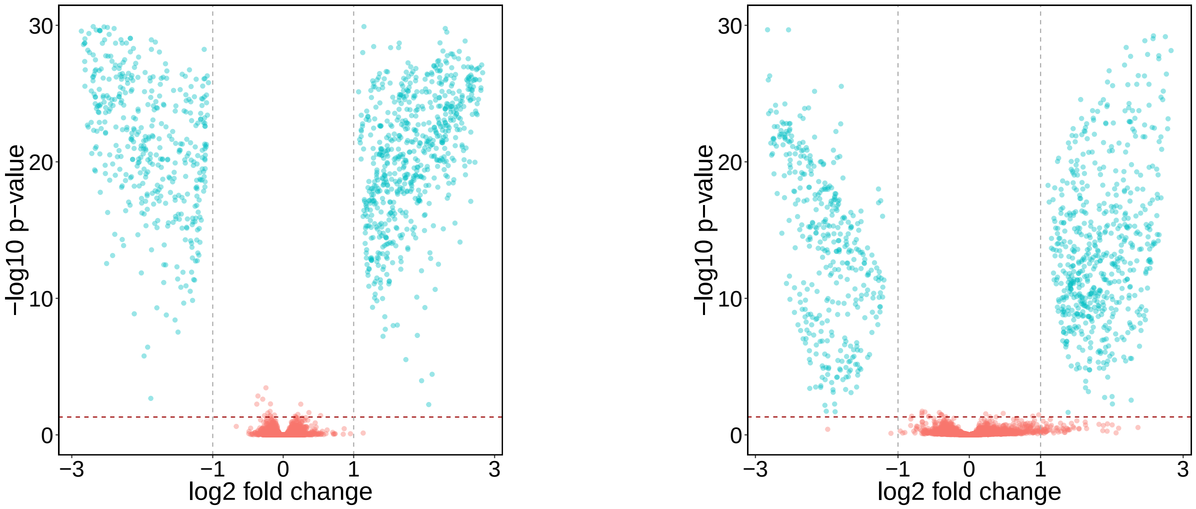


Figure S1: Volcano plots showing differentially expressed genes resulting from simulated data (20 control and 20 case samples) using datasets generated from GSE13355 (left) and GSE54456 (right). The blue points represent genes that were differentially expressed and the red points represent non-differentially expressed genes. The vertical dotted lines indicate the log fold change thresholds that were considered significant. The red horizontal line indicates the p-value cutoff 0.05. The p-values were obtained by performing differential expression analysis using the *limma* R package.

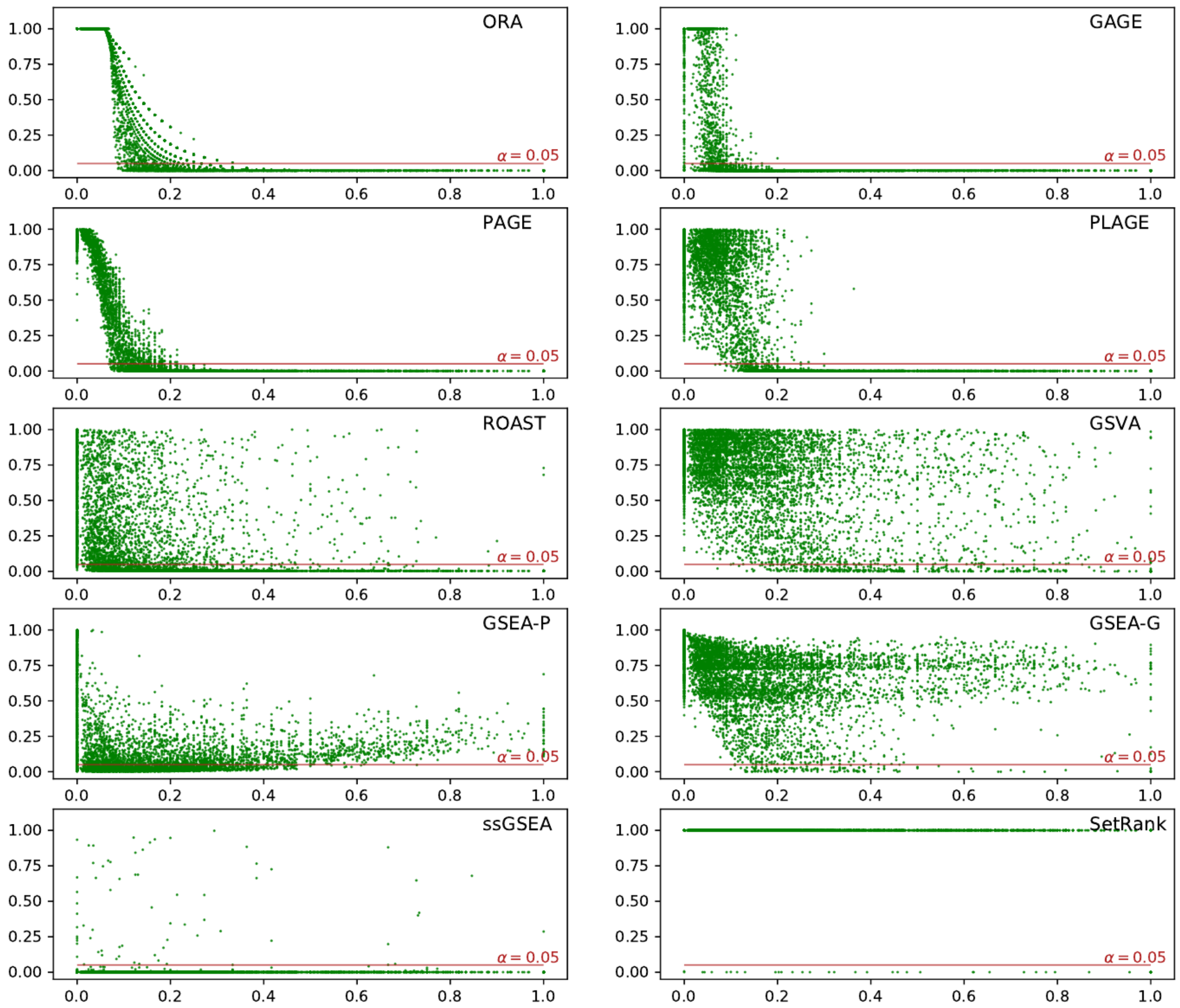


Figure S2: Scatter plots of the relationship between gene set coverage (x-axis) and the statistical significance (adjusted p-value) of the results of each method (y-axis) using dataset generated from GSE13355. Each point in green represents a gene set. The red line shows a p-value cutoff of $\alpha = 0.05$. Since SetRank only returned statistically significant results (points under the red line), we assign a p-value of 1 to visualize the coverage scores for non-significant results. Note that no cut-off value of $\gamma$ is applied in any of these scatter plots.

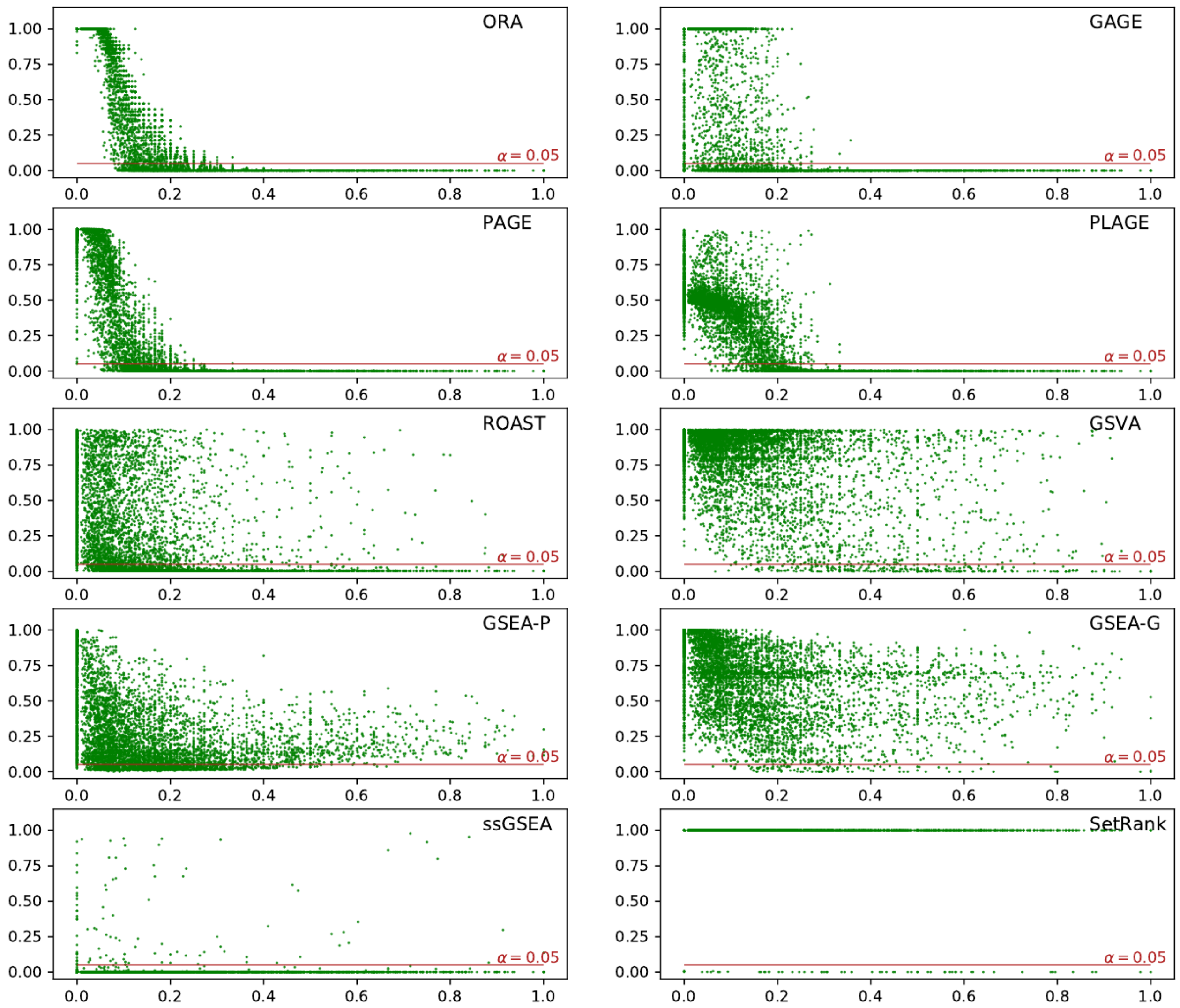


Figure S3: Scatter plots of the relationship between gene set coverage (x-axis) and the statistical significance (adjusted p-value) of the results of each method (y-axis)using dataset generated from GSE54456. Each point in green represents a gene set. The red line shows a p-value cutoff of $\alpha = 0.05$. Since SetRank only returned statistically significant results (points under the red line), we assign a p-value of 1 to visualize the coverage scores for non-significant results. Note that no cut-off value of $\gamma$ is applied in any of these scatter plots.

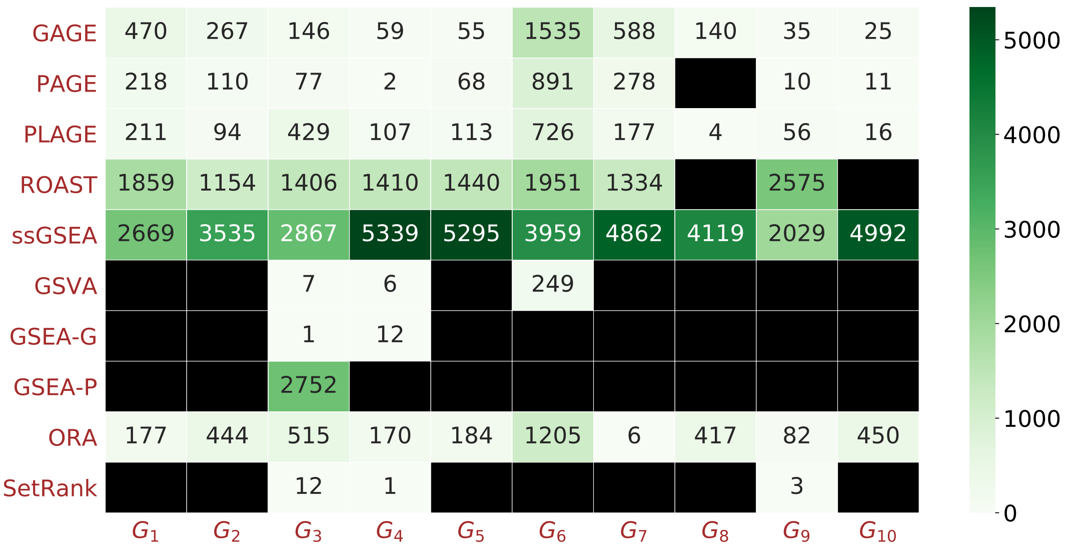


Figure S4: Heat map of the rank of the 10 target gene sets as reported by each method using the synthesized dataset generated from GSE13355. The results of each method were sorted based on the adjusted p-values (smallest to largest); the rank of each target gene set was determined as its rank in the sorted list. The rank is then recorded in each cell as well as encoded by the colour of the cell, where a darker green represents a gene set being later in the sorted list. A black cell with no number shows that the adjusted p-value was not less than $\alpha = 0.05$.

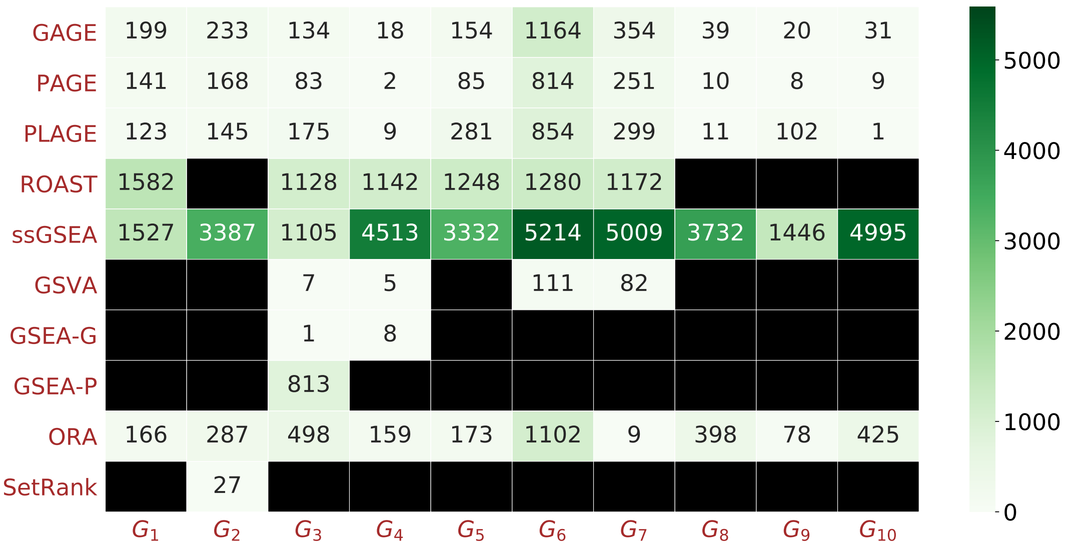


Figure S5: Heat map of the rank of the 10 target gene sets as reported by each method using the synthesized dataset generated from GSE54456. The results of each method were sorted based on the adjusted p-values (smallest to largest); the rank of each target gene set was determined as its rank in the sorted list. The rank is then recorded in each cell as well as encoded by the colour of the cell, where a darker green represents a gene set being later in the sorted list. A black cell with no number shows that the adjusted p-value was not less than $\alpha = 0.05$.

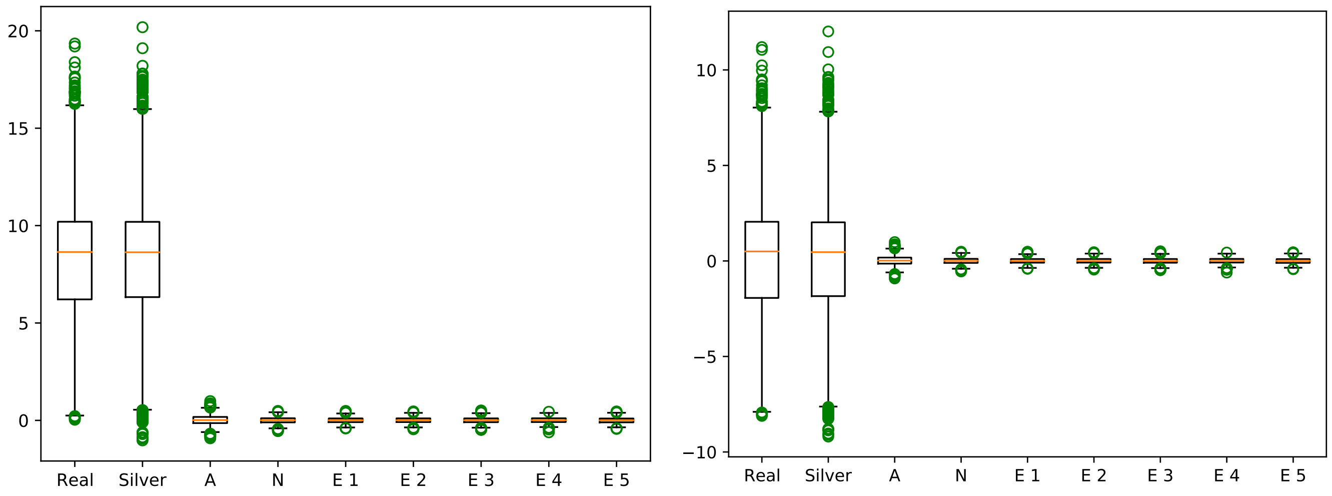


Figure S6: Distributions of the expression values of different synthesized datasets (left), including a dataset synthesized with Silver, compared to the distribution of expression values from a real dataset (GSE54456). The datasets labeled “E 1” to “E 5” were introduced by Efron and Tibshirani [1]. The dataset labeled “N” was introduced by Nam and Kim [2], and the dataset labeled “A” was introduced by Ackermann and Strimmer [3]. While the dataset generated by Silver closely mirrors the real dataset (GSE54456), the other simulated datasets show a substantial difference with the real data. Also, this difference is not due to a constant shift in average expression values, as illustrated by the box plot representing the distribution of centered average expression values for all datasets (right).

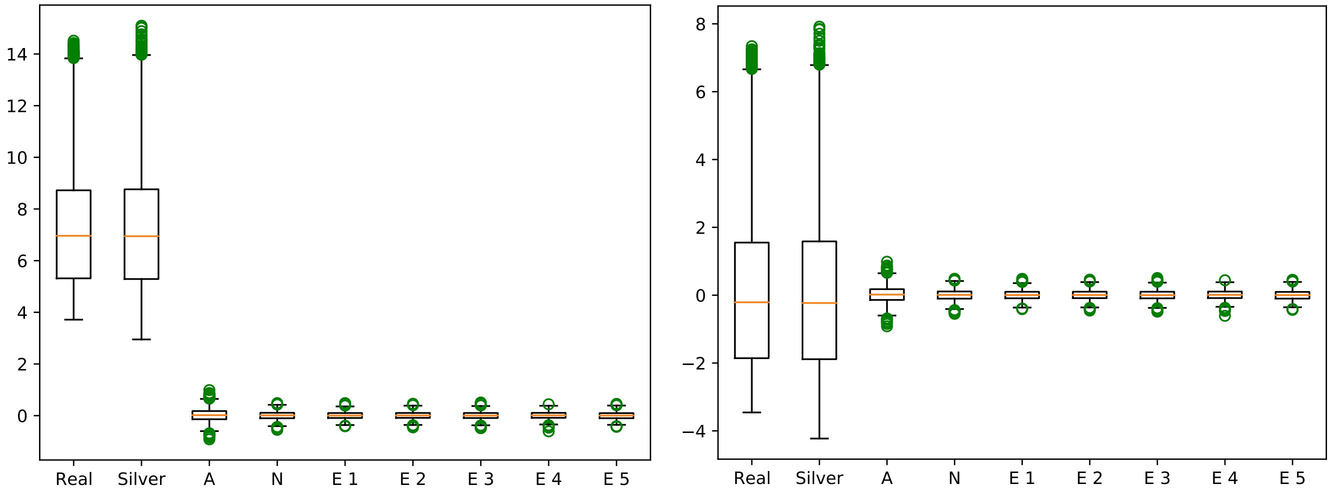


Figure S7: Distributions of the expression values of different synthesized datasets (left), including a dataset synthesized with Silver, compared to the distribution of expression values from a real dataset (GSE13355). The datasets labeled “E 1” to “E 5” were introduced by Efron and Tibshirani [1]. The dataset labeled “N” was introduced by Nam and Kim [2], and the dataset labeled “A” was introduced by Ackermann and Strimmer [3]. While the dataset generated by Silver closely mirrors the real dataset (GSE13355), the other simulated datasets show a substantial difference with the real data. Also, this difference is not due to a constant shift in average expression values, as illustrated by the box plot representing the distribution of centered average expression values for all datasets (right).

Table S1: The sensitivity (TPR) and specificity (TNR) of gene set analysis methods for data simulated from GSE13355.

| | $\gamma = 0.1$ | | $\gamma = 0.3$ | | $\gamma = 0.5$ | | $\gamma = 0.9$ | | $\gamma = 0.99$ | |
|---|---|---|---|---|---|---|---|---|---|---|
| Method | TNR | TPR | TNR | TPR | TNR | TPR | TNR | TPR | TNR | TPR |
| GAGE | 0.75 | 0.99 | 0.40 | 1.00 | 0.35 | 1.00 | 0.33 | 1.00 | 0.33 | 1.00 |
| GSEA-G | 1.00 | 0.04 | 0.98 | 0.06 | 0.98 | 0.03 | 0.98 | 0.17 | 0.98 | 0.20 |
| GSEA-P | 0.57 | 0.50 | 0.47 | 0.19 | 0.49 | 0.01 | 0.53 | 0.00 | 0.53 | 0.00 |
| GSVA | 1.00 | 0.11 | 0.98 | 0.25 | 0.95 | 0.29 | 0.94 | 0.50 | 0.94 | 0.52 |
| PAGE | 0.97 | 0.89 | 0.60 | 1.00 | 0.53 | 1.00 | 0.48 | 1.00 | 0.48 | 1.00 |
| PLAGE | 1.00 | 0.76 | 0.70 | 1.00 | 0.62 | 1.00 | 0.57 | 1.00 | 0.57 | 1.00 |
| Roast | 0.80 | 0.71 | 0.58 | 0.82 | 0.54 | 0.85 | 0.51 | 0.94 | 0.51 | 0.92 |
| ssGSEA | 0.01 | 0.99 | 0.01 | 0.99 | 0.01 | 0.98 | 0.01 | 0.97 | 0.01 | 0.96 |
| SetRank | 1.00 | 0.01 | 1.00 | 0.02 | 1.00 | 0.04 | 0.99 | 0.22 | 0.99 | 0.28 |
| ORA | 0.99 | 0.69 | 0.76 | 0.91 | 0.50 | 1.00 | 0.44 | 1.00 | 0.44 | 1.00 |

Table S2: The sensitivity (TPR) and specificity (TNR) of gene set analysis methods for data simulated from GSE54456.

| | $\gamma = 0.1$ | | $\gamma = 0.3$ | | $\gamma = 0.5$ | | $\gamma = 0.9$ | | $\gamma = 0.99$ | |
|---|---|---|---|---|---|---|---|---|---|---|
| Method | TNR | TPR | TNR | TPR | TNR | TPR | TNR | TPR | TNR | TPR |
| GAGE | 0.86 | 0.82 | 0.59 | 1.00 | 0.52 | 1.00 | 0.48 | 1.00 | 0.48 | 1.00 |
| GSEA-G | 1.00 | 0.03 | 0.99 | 0.05 | 0.98 | 0.04 | 0.98 | 0.14 | 0.98 | 0.23 |
| GSEA-P | 0.91 | 0.24 | 0.82 | 0.15 | 0.82 | 0.02 | 0.83 | 0.00 | 0.83 | 0.00 |
| GSVA | 1.00 | 0.09 | 0.99 | 0.23 | 0.98 | 0.33 | 0.95 | 0.53 | 0.95 | 0.52 |
| PAGE | 0.99 | 0.81 | 0.67 | 1.00 | 0.59 | 1.00 | 0.55 | 1.00 | 0.54 | 1.00 |
| PLAGE | 1.00 | 0.55 | 0.85 | 1.00 | 0.75 | 1.00 | 0.70 | 1.00 | 0.69 | 1.00 |
| Roast | 0.76 | 0.67 | 0.57 | 0.72 | 0.53 | 0.74 | 0.51 | 0.92 | 0.51 | 0.92 |
| ssGSEA | 0.02 | 0.98 | 0.02 | 0.98 | 0.02 | 0.97 | 0.02 | 0.94 | 0.02 | 0.96 |
| SetRank | 1.00 | 0.02 | 1.00 | 0.04 | 0.99 | 0.06 | 0.99 | 0.14 | 0.99 | 0.17 |
| ORA | 0.99 | 0.67 | 0.63 | 0.99 | 0.53 | 1.00 | 0.47 | 1.00 | 0.47 | 1.00 |

Table S3: Comparison of the distribution of average expression values of dataset synthesized by Silver and that of other simulated datasets used for the evaluation of gene set analysis methods. Datasets labeled “E 1” to “E 5” have been introduced by Efron and Tibshirani [1], the dataset labeled “N” has been introduced by Nam and Kim [2], and the dataset labeled “A” has been introduced by Ackermann and Strimmer [3]. To make sure that the differences between the distribution of average expression values are not due to a constant shift in expression values, all datasets have been centered prior to conducting two-sample KolmogorovSmirnov tests. As the results of KolmogorovSmirnov tests indicate, the distribution of average expression of datasets simulated by Silver shows no statistically significant difference with that of real data (GSE13355). However, there are significant differences between the average expression values of the other datasets with that of the real data.

| Dataset | Statistic | p-value |
|---|---|---|
| Silver | 0.007 | 0.674 |
| E 1 | 0.477 | <1.0E-105 |
| E 2 | 0.477 | <1.0E-105 |
| E 3 | 0.476 | <1.0E-105 |
| E 4 | 0.474 | <1.0E-105 |
| E 5 | 0.478 | <1.0E-105 |
| A | 0.447 | <1.0E-105 |
| N | 0.472 | <1.0E-105 |

Table S4: Comparison of the distribution of average expression values of dataset synthesized by Silver and that of other simulated datasets used for the evaluation of gene set analysis methods. Datasets labeled "E 1" to "E 5" have been introduced by Efron and Tibshirani [1], the dataset labeled "N" has been introduced by Nam and Kim [2], and the dataset labeled "A" has been introduced by Ackermann and Strimmer [3]. To make sure that the differences between the distribution of average expression values are not due to a constant shift in expression values, all datasets have been centered prior to conducting two-sample KolmogorovSmirnov tests. As the results of KolmogorovSmirnov tests indicate, the average expression of datasets simulated by Silver shows no statistically significant difference with that of real data (GSE54456). However, there are significant differences between the average expression values of the other datasets with that of the real data.

| Dataset | Statistic | p-value |
|---|---|---|
| Silver | 0.012 | 0.212 |
| E 1 | 0.517 | <1.0E-112 |
| E 2 | 0.517 | <1.0E-112 |
| E 3 | 0.512 | <1.0E-112 |
| E 4 | 0.517 | <1.0E-112 |
| E 5 | 0.518 | <1.0E-112 |
| A | 0.460 | <1.0E-112 |
| N | 0.514 | <1.0E-112 |

# References


[1] Efron, B., Tibshirani, R.: On testing the significance of sets of genes. The Annals of Applied Statistics **1**(1), 107–129 (2007)

[2] Nam, D., Kim, S.-Y.: Gene-set approach for expression pattern analysis. Briefings in Bioinformatics **9**(3), 189–197 (2008)

[3] Ackermann, M., Strimmer, K.: A general modular framework for gene set enrichment analysis. BMC Bioinformatics **10**(1), 47 (2009)
